# Postgraduate medical education in fragile conflict-affected settings: A scoping review

**DOI:** 10.1371/journal.pgph.0005749

**Published:** 2026-04-22

**Authors:** Abd Arrahman Alomar, Ceri Evans, Athanasios Hassoulas, Liz Forty

**Affiliations:** School of Medicine, Cardiff University, Cardiff, United Kingdom; Yale University School of Medicine, UNITED STATES OF AMERICA

## Abstract

Postgraduate medical education (PGME) is a vital pillar of health system resilience, yet it faces catastrophic disruption in Fragile Conflict-Affected settings. This review maps the fragmented body of evidence (current practices, challenges, quality indicators, and accreditation mechanisms), synthesizes insights, and highlights research and policy gaps within PGME programs in these environments. This scoping review was guided by Arksey & O’Malley, Levac et al., and PRISMA-ScR methodological frameworks. A comprehensive search was run across academic databases with additional searches of relevant organizational websites, and library catalogues to uncover grey literature. Elicit (AI) was used as a complementary search tool. The search included studies published between January 2015 and August 2025. Thematic analysis was applied to synthesize the evidence. Of 252 unique records identified, 32 met the inclusion criteria. In terms of geographical location, the countries most frequently reported on were Syria and Ukraine, with the majority of studies taking place over the last three years. Four primary themes were identified: Current Practices and Adaptations, Challenges, Quality and Accreditation, and Innovations and Resilience. Studies and reports from Syria, Ukraine, and Sudan highlighted divergent adaptive pathways, from institutional resilience and frontline training to severe fragmentation and reliance on external support. Consistently, the body of literature is scarce due to systemic barriers (brain drain, research destruction, ethical hurdles). Quality and accreditation emerged as the weakest pillar, often relying on subjective evaluations. PGME in FCA settings demonstrates adaptability through innovations like virtual curricula and diaspora partnerships, yet it is characterized by fragmented evidence and a critical lack of quality assurance. Six recommendations are proposed, focused on institutionalizing resilience models, strengthening program legitimacy through hybrid, internationally benchmarked accreditation [World Federation for Medical Education and Accreditation Council for Graduate Medical Education standards], and integrating mental health and peacebuilding competencies to ensure long-term health system recovery and stability.

## Introduction

Postgraduate medical education (PGME) is a cornerstone of health system resilience, ensuring that physicians acquire advanced competencies to meet population health needs [1]. In stable contexts, PGME is supported by structured curricula, robust mentorship, accreditation processes, and institutional governance [2]. Fragile Conflict-Affected (FCA) settings are characterized by disrupted infrastructure, limited resources, and a high burden of disease. These issues profoundly impact healthcare delivery and medical training, where the aforementioned structural supports collapse under the strain of violence, politicization, and displacement.

The erosion of PGME not only compromises physician training but also undermines long-term health system recovery [1,3]. However, PGME also presents unique opportunities.

Conflicts in Syria, Ukraine, Sudan, Palestine, Afghanistan, and other fragile regions illustrate the multifaceted challenges faced by PGME. Universities and hospitals are attacked or occupied, faculty and trainees are displaced, injured, or even killed, and health systems become fragmented. Conflicts have also generated innovations: diaspora-supported education, virtual curricula, hybrid learning, and ad hoc advanced trauma training.

Global scoping reviews confirm recurring patterns, disrupted curricula, declining trainee wellness, and fragile reliance on partnerships, while revealing substantial knowledge gaps which require additional research and documentation efforts [4,5]. Recent studies from Lebanon, Syria, Ukraine, and Sudan further confirm these patterns, highlighting that PGME programs in conflict zones adjust to instability by modifying curricula and incorporating technology to sustain training despite frequent resource and infrastructure setbacks [1,6–8].

This scoping review encompasses postgraduate medical education across diverse conflict zones and humanitarian crises of the 21st century. Millions of people have been displaced and essential services, including health care, severely disrupted. Such protracted conflicts have left an indelible mark on medical education systems globally. [1,3,4,7].

Such conflicts have severely disrupted medical education, leading to critical shortages of healthcare professionals. For example, since March 2011 through February 2024, Physicians for Human Rights (PHR) corroborated 604 attacks on 400 separate facilities in Syria and documented the killing of 949 medical personnel. The highest percentage killed were doctors (31%), followed by nurses (23%) and paramedics (23%) [9]. These conflicts result in the forced migration of students and faculty members (brain drain). For example, half of the 31,000 reported physicians in Syria have been displaced and by 2013, 70% of the health workforce had left the country, with hundreds more incarcerated or tortured [10,11]. Fragile conflict affected settings face significant declines in the quality of medical training. The challenges of providing education in non-government-controlled areas are well-documented [10,11], with major concerns including limited access to resources, teaching staff, and clinical environments. However, humanitarian organizations are instrumental in establishing and supporting PGME programs globally [1,7,8,11].

Despite the urgency of the issue, the body of literature on PGME in FCA settings remains remarkably limited. Several reasons explain this scarcity. First, research infrastructure is often destroyed or severely weakened during wars, preventing systematic data collection and publication [11,12]. Second, faculty and researchers themselves are displaced, leaving few qualified investigators to document experiences [13]. Third, health priorities in emergencies tend to focus on immediate life-saving service delivery rather than educational continuity, relegating PGME research to the background [4]. Fourth, ethical and logistical barriers — such as limited access, difficulties in obtaining Institutional Review Board (IRB) approvals, and safety risks — complicate prospective studies [14]. Finally, much knowledge remains in the form of grey literature (non-governmental organization “NGO” reports, policy briefs, workshop proceedings), which is underrepresented in academic databases [1,10].

As a result, although the disruption of PGME has profound long-term implications, it has received far less attention compared to undergraduate medical education or immediate humanitarian health interventions. Understanding the recurring themes and patterns within PGME programs in such environments is crucial for developing effective interventions, policies, and support systems.

Therefore, this scoping review aims to map the fragmented body of evidence on PGME in FCA settings (in terms of practices, challenges, quality indicators, and accreditation), synthesize insights across regions, and highlight research and policy gaps.

## Methods

### Study design

Since this topic (PGME in FCA settings) is likely to have limited, heterogeneous (including grey literature), and scattered evidence, a scoping review is deemed the most appropriate approach to map the extent, range and nature of the literature, summarize the findings, and identify possible gaps in the literature.

The study followed the methodological framework for scoping reviews outlined by Arksey & O’Malley [15], further refined by Levac et al. [16], and aligned with the PRISMA Extension for Scoping Reviews (Tricco et al, 2018) [17] considering the implications of the PRISMA 2020 statement where applicable (S1 File).

### Search strategy

#### Search concepts and terms.

Key search concepts included ‘medical education’, ‘postgraduate medical education’ and ‘conflict settings’. These concepts include alternative terms and abbreviations, and this led to the revised search terms used. Key words included: medical, education, training, postgraduate, residency, fellowship, conflict, war.

#### Date range.

A search was conducted for information from January 2015 to August 2025. The start date was chosen to ensure the review captured literature relevant to recent, ongoing, and modern conflicts affecting PGME.

#### Sources of evidence.

Evidence was gathered from a diverse array of published literature including: Primary research, for example, descriptive, observational and experimental studies; and secondary research, for example, systematic reviews, scoping reviews, meta-analyses, perspective pieces such as editorials, commentaries and expert opinion pieces. Grey literature sources, for example, policy documents, narrative reports, guidelines, and commentaries, were also included. As many significant insights related to FCA settings remain unpublished, the inclusion of grey literature is crucial in minimizing publication bias and capturing practice and policy shifts.

An experienced librarian supported the development of the search strategy. As illustrated in S1 Fig, a systematic search was conducted across:

1 Academic/published literature databases

Five databases were searched: Medline (Ovid), Scopus, ProQuest Education Database, ProQuest Dissertations & Theses Global, and PubMed. (S2 Fig, S3 Fig, S4 Fig) Database searches were supplemented by full-text documents provided for analysis.

2 Other methods (including grey literature sources)

Four searches were conducted: Elicit, Overton, Syrian Board of Medical Specialties (SBOMS) and Syrian American Medical Society (SAMS) Reports.

The databases were searched on 4 August 2025, and the other methods’ searches were run on 19 August 2025.

### Eligibility Criteria and study selection

Records of various databases and other different sources were identified, article titles and abstracts/summaries were screened, and only included if they met the following criteria:

- Focused on PGME in FCA or post-conflict settings.
- Focused on training practices, challenges, quality, accreditation, innovations, or resilience strategies.
- Focused on medicine, dentistry, public health, or surgical subspecialties.
- Written in English.
- Conducted during the last ten years.

This meant that studies focusing solely on undergraduate medical education were excluded.

### Data extraction and Charting

A standardized charting template was applied to all included articles to extract: author, year of publication, title, DOI, geographical location, research type and study design, setting, population, and key findings. This systematic approach allowed for identification of distinct key concepts and the gathering of empirical evidence directly from the dataset.

### Data analysis

The study utilized a hybrid deductive and inductive coding approach allowing the use of existing frameworks to code known concepts whilst remaining open to discovering new concepts and insights.

The first author has nine years’ experience in managing PGME programs in complex humanitarian contexts like Northwest Syria. This provided a crucial and realistic lens to capture subtle critical insights from grey literature that academic sources often miss due to publication barriers in war zones. In addition, this approach moved beyond formal challenges to identify underlying systemic barriers requiring policy intervention, as well as ensuring that identified policy gaps (like the crucial role of diaspora engagement and virtual education) were meaningful and actionable within the field context.

Using the six-phase guide to thematic analysis outlined by Braun and Clarke [18], initial deductive codes, informed by the existing literature and field experience of the first author, were applied to the entire dataset. New codes were created inductively, where the data did not fit into the predetermined codes. Following the deductive and inductive coding, codes were collated into potential themes which were iteratively reviewed and refined to form the final themes.

## Results

### Selection of evidence sources

**Fig 1:**
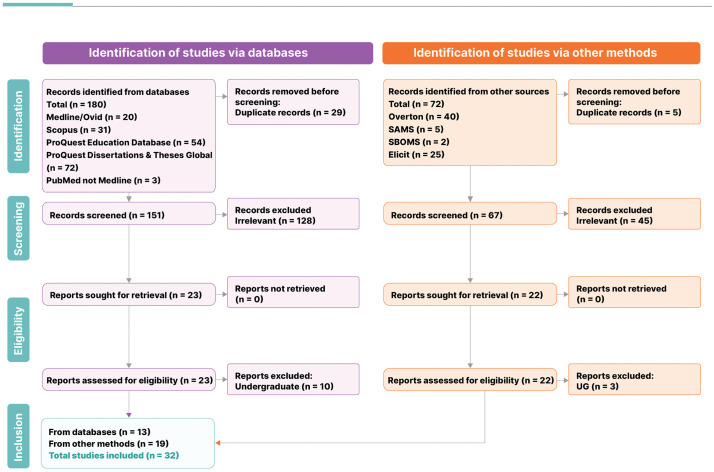
PRISMA FLOW Diagram illustrating the scoping review study selection process.

Fig 1 presents the PRISMA flow diagram detailing the search outcomes, the number of sources assessed, and the final selection for inclusion in this scoping review. A total of 180 unique published articles were identified from Medline (Ovid), Scopus, ProQuest Education Database, ProQuest Dissertations and Theses Global, and PubMed (not Medline) databases. A total of 72 unique sources were identified via other methods. A total of 173 sources were excluded following an initial screening of the titles and abstracts/summaries because they did not report on the topic of interest. In total, 45 full-text records were retrieved. Of these, 13 studies focusing solely on undergraduate medical education were excluded. The final number of sources included in the analysis was 32.

### Characteristics of evidence

Table 1 presents key attributes of the 32 sources including the key reporting theme(s) of the source.

**Table 1:** Summary of ScR included sources.

Author	Year	Title	Geographic Location	Research type and Study Design	Reporting Theme(s)
Abu Aagla et al.	2025	Evaluating Sudanese surgical residents’ perception of the surgical theatre learning environment using the STEEM questionnaire	Sudan	Primary Research: Observational Cross Sectional Study	Challenges; Current Practices and Adaptations; Innovations and Resilience
Bdaiwi et al.	2023	Impact of armed conflict on health professionals’ education and training in Syria: a systematic review	Syria	Secondary Research: Systematic Review	Challenges; Innovations and Resilience
Bdaiwi et al.	2023	Medical education system (re)building in a fragile setting: Northwest Syria as a case study	Syria	Primary Research: Descriptive Study	Challenges; Innovations and Resilience; Quality and Accreditation
Chandradasa and Champika	2019	Subspecialisation in Postgraduate Psychiatry and Implications for a Resource-Limited Specialised Child and Adolescent Mental Health Service	Sri Lanka	Secondary Research: Perspective Piece	Challenges
Dobiesz et al.	2022	Maintaining health professional education during war: A scoping review	Multi-site	Secondary Research: Scoping Review	Challenges; Current Practices and Adaptations; Innovations and Resilience; Quality and Accreditation
Dzhemiliev et al.	2023	Patient Care and Surgical Training During Armed Conflict: Experiences and Perspectives of Surgical Residents in Ukraine	Ukraine	Secondary Research: Perspective Piece	Current Practices and Adaptations; Innovations and Resilience
Dzhemiliev et al.	2024	Strengths and opportunities for improvement in surgical education in Ukraine: A qualitative study	Ukraine	Primary Research: Observational Descriptive Study	Challenges; Current Practices and Adaptations; Innovations and Resilience; Quality and Accreditation
Gaarder and Naess	2024	Invited commentary to strengths and opportunities for improvement in surgical education in Ukraine: A qualitative Study	Ukraine	Secondary Research: Perspective Piece	Challenges; Quality and Accreditation
Hameed et al.	2018	Can online distance learning improve access to learning in conflict zones? The Oxford psychiatry in Iraq (OxPIQ) experience.	Iraq	Primary Research: Descriptive Study	Innovations and Resilience
Hanafi et al.	2021	Medical research conduct and publication during Higher Education in Syria: Attitudes, barriers, practices and possible solutions	Syria	Primary Research: Observational Descriptive Cross-Sectional Study	Challenges; Innovations and Resilience
Hanafi et al.	2025	Improving Academic Writing in a Low-Resource Country: A Systematic Examination of Online Peer-Run Training	Syria	Primary Research: Experimental Study	Challenges; Innovations and Resilience; Quality and Accreditation
Hodgetts et al.	2020	Twenty-five years on: revisiting Bosnia and Herzegovina after implementation of a family medicine development program	Bosnia	Primary Research: Observational Study	Challenges; Current Practices and Adaptations
Kakoma	2016	Postgraduate and research programmes in Medicine and Public Health in Rwanda: an exciting experience about training of human resources for health in limited resources country	Rwanda	Secondary Research: Perspective Piece	Current Practices and Adaptations; Innovations and Resilience
Kaspruk et al.	2025	Directions for Improving the quality of postgraduate education of physicians	Ukraine	Primary Research: Descriptive Study	Innovations and Resilience; Quality and Accreditation
Lamb	2017	Driven out, but Ukraine’s universities are not down	Ukraine	Secondary Research: Perspective Piece	Current Practices and Adaptations; Innovations and Resilience
Mahgoub et al.	2024	Dental education amid armed conflict in Sudan: Unveiling the impact on training	Sudan	Primary Research: Descriptive Study	Challenges; Current Practices and Adaptations; Innovations and Resilience
Mitchell et al.	2023	Surgical Training for Civilian Surgeons Interested in Humanitarian Surgery: A Scoping Review	Multi-site	Secondary Research: Scoping Review	Current Practices and Adaptations; Quality and Accreditation
Msheik El Khoury et al.	2025	Graduate Medical Education in Lebanon: Challenges, Support, and Adaptation Amid the Compounding Crises	Lebanon	Primary Research: Descriptive Cross-Sectional Study	Challenges; Current Practices and Adaptations
Naal et al.	2023	Evaluating a research training programme for frontline health workers in conflict-affected and fragile settings in the middle east	Middle East and North Africa	Primary Research: Descriptive Study	Challenges; Current Practices and Adaptations; Quality and Accreditation
Nazzal et al.	2025	Towards researcher physicians in Palestine: resident doctors’ perceptions, practices and barriers	Palestine (West Bank)	Primary Research: Descriptive Cross-Sectional Study	Quality and Accreditation; Challenges
Omer et al.	2025	The Impact of the Ongoing Armed Conflict on Resident’s Neurosurgical Training and Practice in Sudan: Challenges, Disruptions, and Potential Support Strategies	Sudan	Primary Research: Descriptive Cross -Sectional Study	Challenges; Innovations and Resilience
Qayumi et al.	2024	Enhancing medical training in conflict zones and remote areas through innovation: introducing the Canadian Virtual Medical University Initiative	Afghanistan	Primary Research: Experimental Study	Challenges; Current Practices and Adaptations; Innovations and Resilience; Quality and Accreditation
SAMS	2023	Mentors’ evaluation report – SBOMS program	Syria	Grey literature: NGO Report	Quality and Accreditation
SAMS	2024	SAMS Monitoring Report-GIZ Project Component 2: Resident Doctors Program from January to March 2024	Syria	Grey literature: NGO Report	Challenges; Current Practices and Adaptations; Innovations and Resilience; Quality and Accreditation
SAMS	2024	SAMS Monitoring Report-GIZ Project Component 4: SBOMS	Syria	Grey literature: NGO Report	Challenges; Current Practices and Adaptations; Innovations and Resilience; Quality and Accreditation
SAMS	2025	Simulation-Based Training for Health Professionals in Syrian Public Hospitals: A National Needs Assessment Study	Syria	Grey literature: NGO Report	Quality and Accreditation
SAMS	2025	Evaluation of Medical Education Programs in Syria Challenges, and Opportunities	Syria	Grey literature: NGO Report	Challenges; Quality and Accreditation
Sbei et al.	2025	Reforming Graduate Medical Education in Syria: A Strategic Framework for Post-Conflict Recovery	Syria	Secondary Research: Perspective Piece	Challenges; Current Practices and Adaptations; Innovations and Resilience; Quality and Accreditation
SBOMS	2025	Programmatic report for the Medical Specialties Project	Syria	Grey literature: NGO Report	Challenges; Current Practices and Adaptations; Innovations and Resilience; Quality and Accreditation
SBOMS	2025	SBOMS Legacy Story of Resilience and Impact – From Emergency Response to Building a Sustainable Healthcare Future in Syria	Syria	Grey literature: NGO Report	Innovations and Resilience
Wandschneider et al.	2024	War and peace in public health education and training: a scoping review	Multi-site	Secondary Research: Scoping Review	Quality and Accreditation; Innovations and Resilience
Yacoubian et al.	2023	Burnout among postgraduate medical trainees in Lebanon Potential strategies to promote wellbeing.	Lebanon	Primary Research: Descriptive Cross-Sectional Study	Challenges

NGO: Non-Governmental Organisation.

A summary of the most common characteristics of the whole dataset can be seen in Table 2

**Table 2:** Summary of the characteristics of the sources included in the scoping review.

Data Variable	Findings
**Timeframe and Recency (Year)**	Spanning a nine-year period from **2016 to 2025**. The most frequent year of publication is **2025** (11 sources)
**Source Type**	Primary Research (15) Secondary Research (10) Grey Literature (7)
**Geographic Location**	**Multi-site** (3 sources), **Syria** (12 sources), **Ukraine** (5 sources), **Sudan** (3 sources), **Lebanon** (2 sources), MENA (1 sources), **Sri Lanka** (1 source), **Afghanistan** (1 source), **Bosnia** (1 source), **Palestine** (1 source), **Rwanda** (1 source), **IRAQ** (1 source)
**Reporting Theme**	**Challenges** (22 mentions), **Quality and Accreditation** (18 mentions), **Innovations and Resilience** (21 mentions), **Current Practices and Adaptations** (16 mentions)

The analysis of the source data reveals a body of literature that is highly current, academically grounded, and intensely focused on the challenges facing health professional education in FCA contexts.

**Fig 2:**
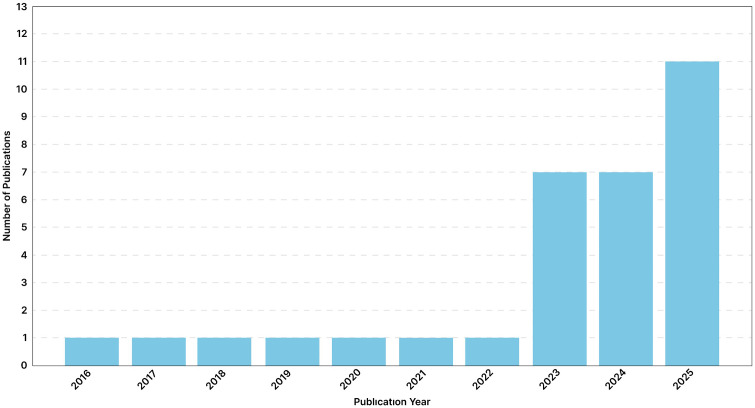
Distribution of included articles by year of publication (N = 32).

The literature spans a recent nine-year period (2016–2025), with a concentration of publications in the most recent three years (2023–25) (Fig 2).

Geographically, the research maintains a broad global perspective but exhibits a clear and specific focus on regions currently or recently affected by conflict. The most frequently mentioned region is Syria due in part to the inclusion of a number of NGO reports from Syria (where the first author has extensive experience in managing PGME programs). In terms of the primary and secondary research articles included in the scoping review, Syria and Ukraine are the most frequently reported geographical locations, suggesting these locations may serve as primary case studies for understanding educational resilience and adaptation in times of crisis.

### Thematic analysis

**Fig 3:**
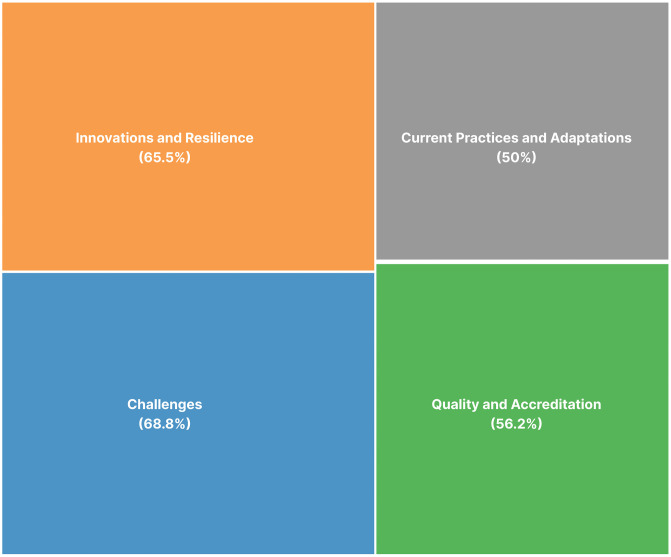
Cross-cutting themes in the included articles (N = 32).

Thematic analysis of the content reported by all the sources included in the scoping review revealed four overarching themes (Fig 3) and 22 codes, shown in Table 3. The two major cross-cutting themes were challenges and innovations & resilience (mentioned in 69% and 65% of the papers, respectively), followed by quality & accreditation and current practices & adaptations (56% and 50%, respectively).

**Table 3:** Summary of Data Analysis (Themes and Codes).

Theme	Codes	Description
**1. Current Practices and Adaptations**	**1.Competency-based or modular curricula** **2. Technology-enhanced learning** 3. Competing authorities* 4. Institutional Resilience* **5. virtual universities** **6.Diaspora Engagement**	Explore how PGME programs adapt to conflict conditions, revealing both shared patterns (adaptation under pressure) and divergent pathways based on institutional strength, technology-enhanced learning, and external support.
**2. Challenges**	**1. Structural** 2. Resource limitations* 3. Political* **4. Faculty** **5. Professional Gaps** **6. Equity and Well-being** 7. Research Disruption*	Detail the multifaceted obstacles faced by PGME in FCA settings, spanning physical, political, workforce, resources, and academic domains.
**3. Quality and Accreditation**	1. Accreditation Gaps* **2. Need for Independent Oversight** 3. Quality indicators* 4. Feasibility of Hybrid Models* **5. Curriculum Deficiencies**	Highlight the consistent weaknesses in quality assurance and the urgent need for robust, independent accreditation mechanisms to ensure the standard of training.
**4. Innovations and Resilience**	1. Technology* 2. Diaspora Engagement* 3. Conflict Medicine* **4. Peacebuilding**	Focus on the positive, adaptive strategies and innovations that emerge from the necessity of operating in FCA environments, leveraging technology and transnational support.

*Deductive Codes

### Current practices and adaptations

Practices include adopting competency-based or modular curricula [8,13,19], and integration of international guidelines [20,21]. Technology-enhanced learning, specifically distance and interactive learning, online mentorship, and virtual teams, was a key adaptation to maintain continuity. For instance, programs in Iraq, Ukraine, and Syria leveraged online platforms to overcome geographical and logistical barriers [11,22,23].

Moreover, Syria illustrated the consequences of fragmentation, where postgraduate training there did not collapse entirely, but instead operated within hospitals governed by competing authorities and hybrid governance models [11].

Ukraine represented a model of institutional resilience where universities that were displaced by war continued to function by shifting rapidly to blended and distance learning models [23], while surgical residents gained crucial hands-on trauma experience in frontline hospitals [24].

In Sudan, with nearly 80% of dental schools attacked, the country was forced to rely heavily on partial online learning and on sending students abroad for advanced placements. Unlike Ukraine, where distance learning was built on existing institutional networks, in Sudan, these alternatives widened inequalities, privileging those with access to international networks or digital connectivity [12].

The establishment of virtual universities in Afghanistan, such as the CVMUI model, made education accessible despite insecurity, and crucially, reduced costs while enhancing the skills base [25]. Rather than serving merely as a stopgap, this approach restructured medical training into a more decentralised, technology-driven system that may outlast the conflict itself [25].

Sri Lanka demonstrated the importance of returning diaspora professionals stepped in to fill critical gaps [26].

### Challenges

PGME in FCA zones faced multifaceted and serious challenges, that compromised both infrastructure and human capital [7].

Universities and hospitals in Sudan, Ukraine, and Syria were directly impacted. The pervasive destruction of training centers, in addition to other aspects of structural damage, such as the lack of simulation labs, further hindered the practical and safe training environment [10,12,27]. Inadequate facilities were also noted in Afghanistan and Ukraine [7].

Resource limitations, including shortages of equipment, supplies, and funding, were widely reported [7]. Economic collapse, such as in Lebanon, led to shortages of medicines and high drug prices. These constraints were significant barriers to the quality and continuity of PGME [6].

The educational process in Syria suffered from politicization, and the contested legitimacy of educational institutions in Ukraine was challenging [11,27].

The faculty/workforce required to maintain the sustainability of these programs rapidly eroded. Faculty shortages and displacement (brain drain) were recurring challenges, with qualified staff being lost or displaced due to conflicts in regions like Syria, Sudan, and Liberia. Dependence on diaspora became a must [2,16,28,29]. Several systemic barriers were consistently raised, such as financial stress (low salaries, delay in paying salaries) and medical equipment shortage.

Professional gaps impacted the quality of training, such as lack of structured curricula, and evaluations in Sudan, the shortage of qualified trainers/supervisors in Syria and the dominance of self-directed learning in Ukraine [27,30].

Issues of equity and well-being obviously emerged. Gender discrimination was a systemic barrier in Ukrainian surgical training. Trainee wellbeing was recognized as a critical issue, with high rates of burnout, psychological distress, and safety concerns reported in Lebanon, Sudan, and other settings, and trainee wellness largely was under-reported and undocumented [27,31].

Weak infrastructure, displacement of faculty, and ethical barriers disrupted research and limited knowledge production [10,14,32].

### Quality and accreditation

Accreditation and quality assurance consistently emerged as areas of documented weakness. The absence of independent accreditation was identified in Ukraine, which led to the dominance of subjective evaluations [27,30].

The imperative of robust and independent oversight was further emphasized in Syria, which underscored the need for an independent, depoliticized, and dedicated council for PGME. Other crucial requirements, such as improving training quality, strengthening faculty development, enhancing research contributions, and drawing from international benchmarks such as the World Federation for Medical Education (WFME) [33] and the Accreditation Council for Graduate Medical Education (ACGME) [34], were deemed essential [1,11]. Quality indicators are measured through structured examinations (e.g., multiple choice tests, OSCEs, logbooks), national and international accreditation standards, stakeholder and patient feedback, and assessments of research engagement. However, quality assurance was often aspirational, with gaps in implementation due to resource constraints and lack of accreditation systems [1,5,21,29,31].

In Bosnia, the 15-year collaborative history demonstrated the practical feasibility of hybrid models, which effectively combined local oversight with international accreditation standards [35].

Systemic weaknesses were also evident in curricula design, specifically in public health dimensions. Curricula tended to focus on trauma and emergencies, while critically neglected fundamental training components related to war prevention and peacebuilding [36].

### Innovations and resilience

Remarkable innovations and demonstrated resilience were notably reported in regions impacted by conflict, which were imperative to sustain and advance medical education.

Technological adaptation was pivotal in maintaining educational continuity in widely disrupted institutions. Virtual curricula in Afghanistan and the adoption of blended learning platforms in Ukraine and Syria ensured didactic mentorship [10,23,25].

Diaspora Engagement was instrumental in providing specialized support. In Sri Lanka, this engagement focused on strengthening the capacities of subspecialties [26], and trainees in Syria benefited from hybrid faculty support in Syria, a model combining remote mentorship with intermittent on-site training during medical missions [1].

Cadre skills were obviously enhanced through Conflict Medicine. In Syria and Ukraine, residents gained extensive trauma skills beyond standard curricula [10,24].

CREEW fellowship program, offered by Global Health Institute (GHI) at the American University of Beirut (AUB), designed for frontline health practitioners in conflict-affected MENA states, directly supported local Research Capacity [14].

Medical training has shown to play a vital Peacebuilding Role**.** Training in South Sudan fostered intergroup trust alongside the development of essential health skills, directly contributed to social cohesion [36].

## Discussion

This scoping review demonstrates both the adaptability of PGME in FCA settings and the fragility of its evidence base. At the global level, the review highlights the persistence of common barriers and the generally underdeveloped state of research in this field [4,5]. The findings of this scoping review, informed by a rigorous methodological approach, yield the following critical discussion points:

### Scarcity of literature and evidence fragmentation

Compared to the vast literature on humanitarian service delivery, PGME has received little scholarly attention. The number of documents underscores the limited evidence base when set against the scale of global conflicts. This scarcity reflects the many barriers to academic production in war zones, including insecurity, displacement, resource shortages, and the prioritization of urgent clinical service over documentation. Mahgoub 2024 [12], for instance, reported that Sudanese dental schools were physically attacked, with libraries and data destroyed, while Naal 2023 [14] showed that even research fellowships in Lebanon and Syria struggled to obtain Institutional Review Board (IRB) approvals. Several systemic reasons explain the paucity of literature. Research Infrastructure (universities, libraries, and laboratories) is frequently destroyed or looted. Similarly, in Syria, fragmentation of universities dismantled capacity for systematic study [11].

Educators and researchers often flee conflict (brain drain), leaving fewer qualified individuals to conduct, supervise, and publish research locally [1,12]. During emergencies, service provision is necessarily prioritized over conducting research. The clinicians focus intensely on saving lives, while documentation of training becomes secondary [4]. The process of obtaining IRB approval is exceedingly difficult in fragile states, due to ethical and logistical barriers, even for fellowships like CREEW operating in relatively safer conditions [14]. As a consequence, grey literature prevails, and many insights remain trapped in NGO reports or workshop notes, rather than being formally captured in peer-reviewed journals [1,10]. Because of the political sensitivities, publishing research on education in contested settings (as in Syria, Ukraine) could harm authors or delegitimize institutions [1,20]. Taken together, these barriers explain why PGME literature is frequently fragmented, often anecdotal, and disproportionately produced by international, rather than local, researchers.

### Adaptation and resilience

This scoping review reveals that experiences across different conflict settings demonstrate that PGME does not collapse uniformly; rather, it adapts through divergent pathways. The experiences of Syria, Ukraine, Sudan, Afghanistan, and Sri Lanka demonstrated the extent to which postgraduate medical education adapts to conditions of conflict and recovery.

The Ukrainian case suggested that when institutional structures remain intact, even if relocated or virtualized, they can continue to deliver meaningful education [23,24]. However, qualitative studies exposed gender disparities, weak mentorship, and accreditation gaps [24,27]. Afghanistan demonstrated how prolonged instability can foster innovation, where virtual curricula such as CVMUI have proven highly cost-effective [25].

Sri Lanka highlighted the importance of diaspora engagement in the aftermath of war, where domestic systems lacked the expertise to develop advanced subspecialties. The diaspora contributions underscored how transnational networks of expertise can play a decisive role in reconstruction, in contrast to the more technology-driven solutions observed in Afghanistan [26].

In Syria, higher education has suffered from severe politicization, faculty shortages, lack of standardized oversight, and huge financial challenges. Furthermore, limited literature and evidence-based training, ineffective evaluation systems, infrastructure deficiencies, and fragmentation created additional load. However, local initiatives, such as SBOMS/SAMS-led postgraduate medical education programs, have emerged [1,11]. These programs demonstrated that committed local leadership can establish quality clinical training in FCA zones, reflected by a remarkable capacity for educational resilience underpinned by exceptional human capital.

Sudan’s experience showed the vulnerability of education systems when infrastructure is destroyed and institutions are directly targeted. Alternatives have been adopted [12]. Across the MENA region, capacity-building fellowships, such as CREEW training program, sought to address structural deficits in research [14].

Collectively, these findings reveal both shared patterns and divergent pathways. All contexts illustrate the capacity of medical education to adapt under pressure, whether through digital innovation, diaspora mobilization, or experiential frontline learning. Yet the nature of adaptation is shaped by the underlying institutional resilience, the degree of infrastructure destruction, and the extent of available external support.

### The imperative of quality and accreditation

The review consistently identifies accreditation and quality assurance as the weakest pillars of PGME in FCA settings, yet also suggests feasible pathways forward. In many contexts, independent or structured accreditation is lacking, with subjective evaluations often prevailing (e.g., Ukraine). This highlights the systemic lack of structured curricula and formal evaluation systems. For contexts like Syria, there is a clear need for an independent, depoliticized accreditation council and the adoption of international benchmarks, such as those from the World Federation for Medical Education (WFME) [33] and the Accreditation Council for Graduate Medical Education (ACGME) [34], to strengthen legitimacy and training quality [1]. SBOMS’s recent efforts in aligning curricula with global standards and adopting tools like Mini-CEX and EPAs reflect this urgent need for structured quality [36–42].The frameworks provided by the WFME and the ACGME can be adapted for FCA settings by prioritizing flexibility, context-specificity, and core quality assurance over rigid adherence to all high-resource setting standards. Both WFME and ACGME provide a structure for quality assurance in medical education. Their adaptation in FCA settings requires a tailored approach to address the unique challenges of instability, resource scarcity, and mass displacement.

Gaps of accreditation in FCA settings may influence patient safety. It creates a vicious cycle where the instability of the setting comprises educational quality, which in turn produces a less competent workforce, further impacting patient safety within an already fragile health system.

The historical 15-year partnership in Bosnia demonstrated the feasibility and success of a hybrid local-international accreditation model, offering a practical framework for other fragile settings to establish legitimacy and quality oversight [34].

Equity and curriculum gaps are well recognized. Quality extends beyond technical skills to include social competencies. The evidence points to critical gaps in trainee well-being, systemic issues like gender discrimination (e.g., Ukrainian surgical training), and the neglect of topics like war prevention and peace promotion within public health curricula [27,36].

### Implications

Despite scarcity, the available evidence underscores resilience and innovation [8]. Diaspora engagement and virtual education models show promise for scalability [22]. Quality assurance and Accreditation remain the weakest pillar [1]. However, the Bosnian hybrid model offers potential pathways for advancement. Furthermore, the integration of gender equity and peacebuilding competencies into curricula is a critical need. Building research capacity is essential, and even under resource constraints, structured mentorship can yield publishable research.

### Limitations

The study encountered several limitations beyond the scarcity of academic literature.

Focus on academic publications is prominent, while the practical insights and grey literature are underrepresented. The dataset, while including grey literature, may still overlook the full range of practical insights, challenges, and solutions known only to non-governmental organizations or practitioners working on the ground in these complex environments.

The limited scope of training levels is apparent as the review explicitly excluded literature focused solely on undergraduate medical education, or continuing professional development that did not have direct relevance to the postgraduate level. This limitation constrains a full understanding of the entire continuum of medical training disruption.

The scoping review methodology was deemed appropriate due to the heterogeneity of evidence, which included empirical studies, case reports, commentaries, and grey literature. While necessary, this heterogeneity can complicate the synthesis and comparison of findings across different settings.

## Conclusion

PGME in FCA settings is marked by disruption, resilience, and innovation, but also by a fragile and fragmented evidence base, as identified by this scoping review. Despite the critical role of PGME in health system recovery, research in FCA settings remains scarce due to destroyed infrastructure, displacement of faculty, and ethical barriers. Yet innovations such as virtual curricula, diaspora partnerships, and research fellowships highlight the adaptability of PGME.

### Recommendations

Moving forward, to enhance postgraduate medical education programs and related support systems in FCA settings, six imperatives are outlined which directly address the aims of this scoping review by translating the mapped evidence and identified gaps into research and actionable policy:

1. **Institutionalize resilience by scaling diaspora and virtual models into structured systems.** This addresses the adaptation challenge and fragmentation observed across settings. The Afghanistan model demonstrated how the establishment of virtual universities made education accessible, reduced costs, and enhanced skills, potentially outlasting the conflict. The Sri Lankan model showed that returning diaspora professionals were decisive in rebuilding subspecialty expertise. SBOMS in Syria listed SAMS, the American University of Beirut, and the Royal College of Emergency Medicine (RCEM) as key international partners for training and curriculum development, showcasing the established reliance on and success of external expertise.
2. **Strengthen legitimacy through independent accreditation and quality assurance.** This directly addresses the lack of robust quality assurance and accreditation encountered in such contexts. Literature on Ukraine noted the lack of independent accreditation and the prevalence of subjective evaluations as a major weakness. The Syrian context specifically highlighted the urgent need for an independent, depoliticized council for PGME, including strengthening faculty development and drawing from international benchmarks. The historical Bosnia partnership demonstrated the feasibility of a successful hybrid local-international accreditation model, proving a pathway forward.
3. **Expand and adapt curricula to include not only emergency response but also prevention, equity, and peace promotion**. This addresses the need to go beyond trauma care to tackle structural issues and documented curriculum deficiencies. The literature exposed gender disparities in surgical training, and public health curricula were found to focus on emergencies but neglect war prevention and peace promotion. In South Sudan, PGME training was noted to have fostered intergroup trust alongside health skills, suggesting a dual role for education in peacebuilding.
4. **Develop quality-driven approaches to meet the international standards,** mainly that of World Federation of Medical Education (WFME) which grant more adaptable and contextualized capabilities. This underpins the quality-driven shift necessary for external recognition and sustainable systems. The need to draw from international benchmarks is explicitly mentioned as a recommendation for the Syrian context. WFME Global Standards are noted as a suitable reference for quality improvement in postgraduate medical education.
5. **Integrate culturally sensitive mental health support for both trainees and faculty within PGME programs**. This addresses the vulnerability of trainees and faculty and the documented gap in wellness support. Research highlights the high stress experienced by trainees during times of instability, where wellness was highlighted as a component that is largely undocumented and often overlooked.
6. **Prioritize resource mobilization and management focusing on training local faculty, improving infrastructure, and ensuring adequate remuneration.** This addresses the pervasive theme of resource scarcity that formed the core barrier across all settings. The review revealed the destruction of hospitals, lack of simulation labs, severe brain drain, and dependence on diaspora as main challenges.

These recommendations are considered as policy-focused output of the study process, converting observations on “what is happening” (the evidence map) and “what is missing” (the gaps) into concrete steps for “what should be done” in future PGME programs in conflict settings.

## Supporting information

S1 File PRISMA Checklist. PRISMA-ScR Checklist.
(DOCX)

S1 Fig Summary of the Search Strategy. (TIF)

S2 Fig Search Strategy for Ovid MEDLINE. (TIF)

S3 Fig Search Strategy for Scopus. (TIF)

S4 Fig Search Strategy for ProQuest Education Database and ProQuest Dissertations and Theses Global. (TIF)
